# Over-the-wire microelectrode catheter in the middle cardiac vein for treating premature ventricular contractions from the posterior-superior process of the left ventricle

**DOI:** 10.1016/j.hrcr.2025.04.004

**Published:** 2025-04-11

**Authors:** Shinichi Harada, Masato Okada, Akinobu Mizutani, Koji Tanaka, Nobuaki Tanaka

**Affiliations:** 1 Cardiovascular Center, Sakurabashi Watanabe Advanced Healthcare Hospital, Osaka, Japan; 2 Department of Cardiology and Nephrology, Mie University Graduate School of Medicine, Tsu, Japan

**Keywords:** Catheter ablation, Microelectrode catheter, Middle cardiac vein, Posterior-superior process of the left ventricle, Premature ventricular contraction

**Key Teaching Points**

• The posterior-superior process of the left ventricle (PSP-LV) is an anatomically complex region that often requires multichamber mapping to identify the origin of premature ventricular contractions (PVCs).
• The over-the-wire microelectrode catheter facilitates access to the middle cardiac vein, enabling the recording of epicardial electrograms that help identify the earliest activation site of PSP-LV PVCs.
• Integrating epicardial electrograms within the middle cardiac vein with right and left endocardial electrograms allows for a 3-dimensional estimation of the PVC origin, ensuring accurate localization in anatomically challenging regions.
• When radiofrequency ablation at the earliest activation site of PSP-LV PVCs is not feasible due to complex anatomy or the risk of atrioventricular block, increasing power settings and extending ablation time from the nearest accessible chamber can be an effective alternative.

## Introduction

The posterior-superior process of the left ventricle (PSP-LV) is a rare but clinically significant origin of premature ventricular contractions (PVCs). The PSP-LV, located at the most inferior and posterior portion of the basal LV, is anatomically adjacent to the inferomedial aspect of the right atrium (RA). Due to its deep and complex anatomic location, catheter ablation of PVCs originating from the PSP-LV (PSP-LV PVCs) is challenging and often requires mapping from multiple chambers. Successful ablation sites in the literature include the RA, LV, and proximal coronary sinus (CS),^1^, ^2^, ^3^ highlighting the variability in accessing and targeting these arrhythmias.

Advancements in electrophysiological tools have expanded the options for mapping and ablating complex PVCs. One such tool, the 2.7F over-the-wire (OTW) microelectrode catheter (EPstar Fix AIV, Japan Lifeline, Tokyo, Japan), offers notable advantages. Designed to advance along a 0.014-inch guidewire, this catheter enables detailed mapping within the coronary venous system. Its small diameter and flexible design allow for navigation into the middle cardiac vein (MCV) and its branches—areas that are typically difficult to access using standard electrode catheters.^4^^,^^5^ Here, we present 2 cases of PSP-LV PVCs that were successfully treated with the novel OTW microelectrode catheter inserted into the MCV.

## Case reports

### Case 1

A 66-year-old man with a history of anterior septal myocardial infarction presented to our hospital with palpitations and exertional dyspnea. A 12-lead electrocardiogram showed frequent monomorphic PVCs with a left bundle branch block morphology, superior axis, notched QRS complex in lead V1, and maximum deflection index (MDI) of 0.50 (Supplemental Figure 1). A 24-hour Holter recording demonstrated a PVC burden of 47%, despite treatment with bisoprolol (2.5 mg daily). Echocardiography revealed a left ventricular ejection fraction of 54% with akinesis in the mid-to-apical segments of the anteroseptal wall. Coronary angiography excluded restenosis or new stenosis. Due to concerns about the negative inotropic effects of antiarrhythmic drugs, they were not prescribed. Instead, catheter ablation was planned for PVC treatment.

**Figure 1:**
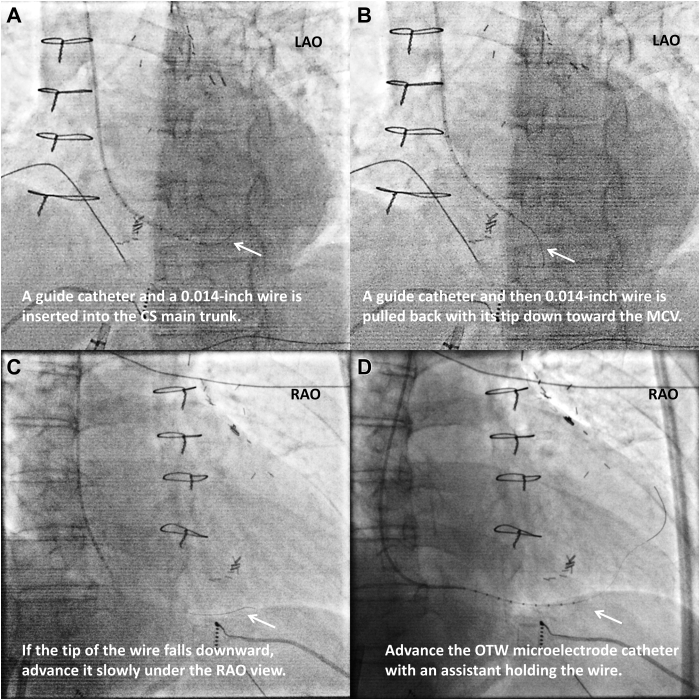
Fluoroscopic images during the catheter insertion procedure. A 5F multipurpose catheter is used to guide the insertion of the over-the-wire (OTW) microcatheter (EPstar Fix AIV, Japan Lifeline, Tokyo, Japan). As a preliminary step, a 0.014-inch guidewire combined with the OTW microelectrode catheter is introduced into the 5F guide catheter outside the body. **A:** After the preparation, the 5F multipurpose catheter is advanced through a sheath into the right atrium with the guidewire ahead of the device. Then, the guidewire and the microelectrode catheter are stored within the 5F multipurpose catheter, which is navigated into the coronary sinus (CS) ostium using its curvature. Subsequently, the 0.014-inch guidewire is advanced into the CS main trunk. **B:** Next. the 5F guide catheter is pulled back into the right atrium, and the wire is also pulled back toward the middle cardiac vein (MCV). **C:** If the wire tip falls downward, it is advanced slowly while confirming its route under the right anterior oblique (RAO) view. **D:** Finally, the OTW microelectrode catheter is advanced along the wire, with an assistant holding it. LAO = left anterior oblique.

After obtaining informed consent, an electrophysiological study was conducted without sedation. Suspecting PSP-LV PVCs, a 0.014-inch guidewire (Runthrough NS, TERUMO, Tokyo, Japan) was initially inserted into the MCV and a 2.7F OTW microelectrode catheter was advanced using a 5F multipurpose catheter as a guide for this microelectrode catheter (Figure 1). We had originally planned to perform activation mapping, however, due to a significant reduction in the clinical PVCs, we instead performed pace mapping using an irrigated-tip catheter (Thermocool Smart Touch STSF, Biosense Webster, Irvine, CA). The highest correlation score (97%) was identified at a site 11 mm caudal to the His bundle area. Although initially presumed to be within the right ventricle, intracardiac echocardiography (CARTOSOUND, Biosense Webster) confirmed its location on the atrial side of the tricuspid annulus.

Fortunately, isoproterenol administration induced clinical PVCs. At the site of the highest pace-map correlation, local electrograms preceded the QRS onset by 24 ms, and unipolar electrograms exhibited a QS pattern. Given the proximity to the atrioventricular node, radiofrequency (RF) energy was cautiously applied using a titration method (10–35 W, 60 seconds), while monitoring the AH interval. The clinical PVCs appeared to be eliminated after the initial RF application, and 2 additional bonus applications were delivered to consolidate the effect.

However, PVCs recurred after further isoproterenol administration. The recurrent PVCs had a similar QRS morphology, but with a wider QRS complex, featuring a notched QS pattern in the inferior leads and an MDI of 0.58. Electroanatomic mapping identified the earliest activation site (EAS) at the roof of the proximal MCV. The local electrograms at this site preceded the QRS onset by 27 ms, with pacing demonstrating a 97% correlation to the recurrent PVC morphology. After confirming the stable generator impedance of the ablation catheter (150–160 Ω) and mild contact force (5–10 *g*), RF energy was applied at this site within the MCV (20 W, 30 seconds), successfully eliminating the PVCs. During the application, impedance gradually decreased to 140–150 Ω, indicating an effective lesion formation. Three additional RF applications were delivered to consolidate the lesion. No further PVCs were observed, even after additional isoproterenol administrations.

**Figure 2:**
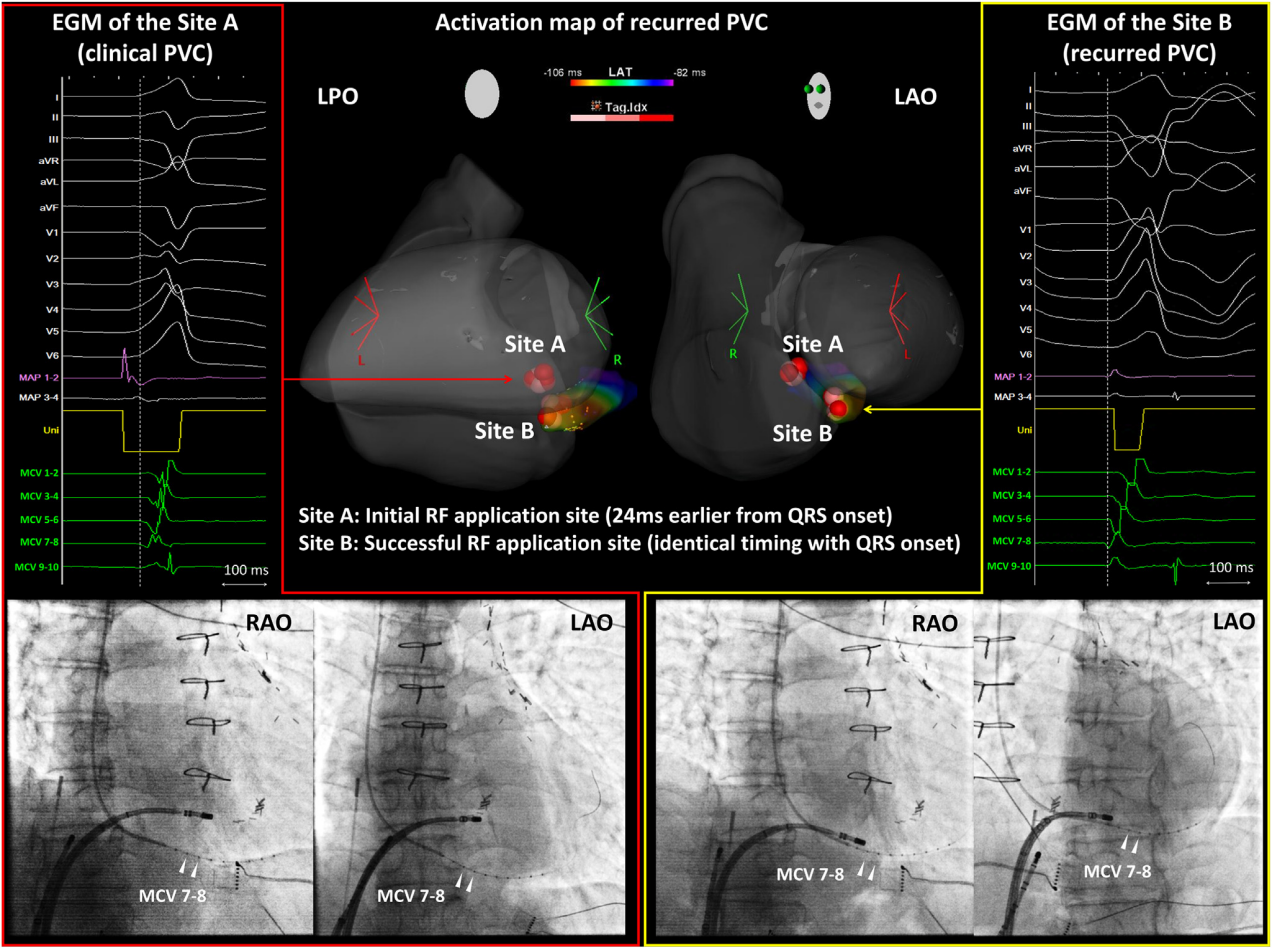
The electroanatomic mapping, intracardiac electrograms (EGMs), and fluoroscopic images of the radiofrequency (RF) energy application sites in case 1. The *red tag* indicates the RF energy application site. Initially, RF energy was applied to the earliest activation site (EAS) at the right atrial septum (*site A*) based on the pace and activation mapping. Although effective, the premature ventricular contraction (PVC) recurred with a slightly different morphology. The EAS then shifted to the proximal electrodes of the microelectrode catheter within the middle cardiac vein (denoted by MCV 7–8). RF energy application at this site (*site B*) successfully eliminated the PVC. LAO = left anterior oblique; LPO = left posterior oblique; RAO = right anterior oblique.

The electroanatomic mapping, intracardiac electrograms, and fluoroscopic images for this case are summarized in Figure 2. A total of 7 RF applications were delivered: 3 targeting the right-sided EAS and 4 directed at the EAS within the MCV. The cumulative energy application time was approximately 120 seconds for the right-sided EAS and 90 seconds for the MCV. A 24-hour Holter recording at 3 months post procedure showed a residual PVC burden of <0.1%, with no need for additional antiarrhythmic therapy. The patient remained free of symptomatic PVCs throughout a 2-year follow-up period.

### Case 2

A 72-year-old man with a history of hypertension was admitted to our hospital with palpitations. A 12-lead electrocardiogram revealed frequent monomorphic PVCs with left bundle branch block morphology, superior axis, a notched QRS complex in lead V1, and an MDI of 0.50 (Supplemental Figure 2). Echocardiography showed normokinesis with a left ventricular ejection fraction of 70%. Coronary angiography confirmed no significant stenosis. The patient had been treated with a class Ic antiarrhythmic agent (flecainide 50 mg twice daily), which resulted in only a modest reduction in PVC burden. Due to persistent symptomatic PVCs despite antiarrhythmic therapy, catheter ablation was performed after obtaining informed consent.

After creating the anatomic contours using the CARTOSOUND intracardiac echocardiography, an electrophysiological study was conducted without sedation. As in case 1, a 2.7F OTW microelectrode catheter was initially inserted into the MCV. The earliest electrograms of the microcatheter preceded the QRS onset by 4 ms. Activation mapping was created using the CARTO3 system and an Optrell catheter (Biosense Webster). The EAS of the right-sided heart was located on the posterior septum near the atrioventricular node. Local electrograms at that site preceded the QRS onset by 8 ms, with unipolar electrograms displaying an rS pattern.

Subsequently, the left ventricle was mapped. The EAS of the left ventricle was identified at the basal septum, contralateral to the right-sided EAS. Local electrograms at the left-sided EAS preceded the QRS onset by 10 ms, with unipolar electrograms exhibiting a QS pattern. However, pacing from this site did not produce a similar QRS morphology (78% correlation), suggesting that the origin was located beneath the endocardial surface, between the right- and left-sided EASs.

Given the proximity of the contralateral site to the atrioventricular node, RF energy was cautiously delivered to the left-sided EAS using a QDOT MICRO Catheter (Biosense Webster). The power was gradually titrated from 15 W to 30 W, while maintaining a mild contact force (5–10 *g*). However, 60 seconds after the RF energy application, a slow junctional rhythm emerged, necessitating the cessation of the energy delivery at that site. RF energy was then applied to the right-sided EAS using a similar titration method (15–30 W). However, repeated junctional rhythm episodes and intermittent atrioventricular block precluded further ablation. In addition, RF energy application within the CS posed a risk due to the high impedance (170–200 Ω) and unstable contact force (10–40 *g*). Therefore, we did not perform it.

Finally, RF energy was reapplied to the left-sided EAS. While closely monitoring for atrioventricular block, the RF energy application was carefully continued, extending the ablation time to 120 seconds (target ablation index = 550–600). During the application, generator impedance gradually decreased from 100 Ω to 90 Ω. This approach successfully eliminated the clinical PVCs. To reinforce the effect, 3 additional RF energy applications (15–30 W, 60–90 seconds) were delivered for lesion consolidation.

**Figure 3:**
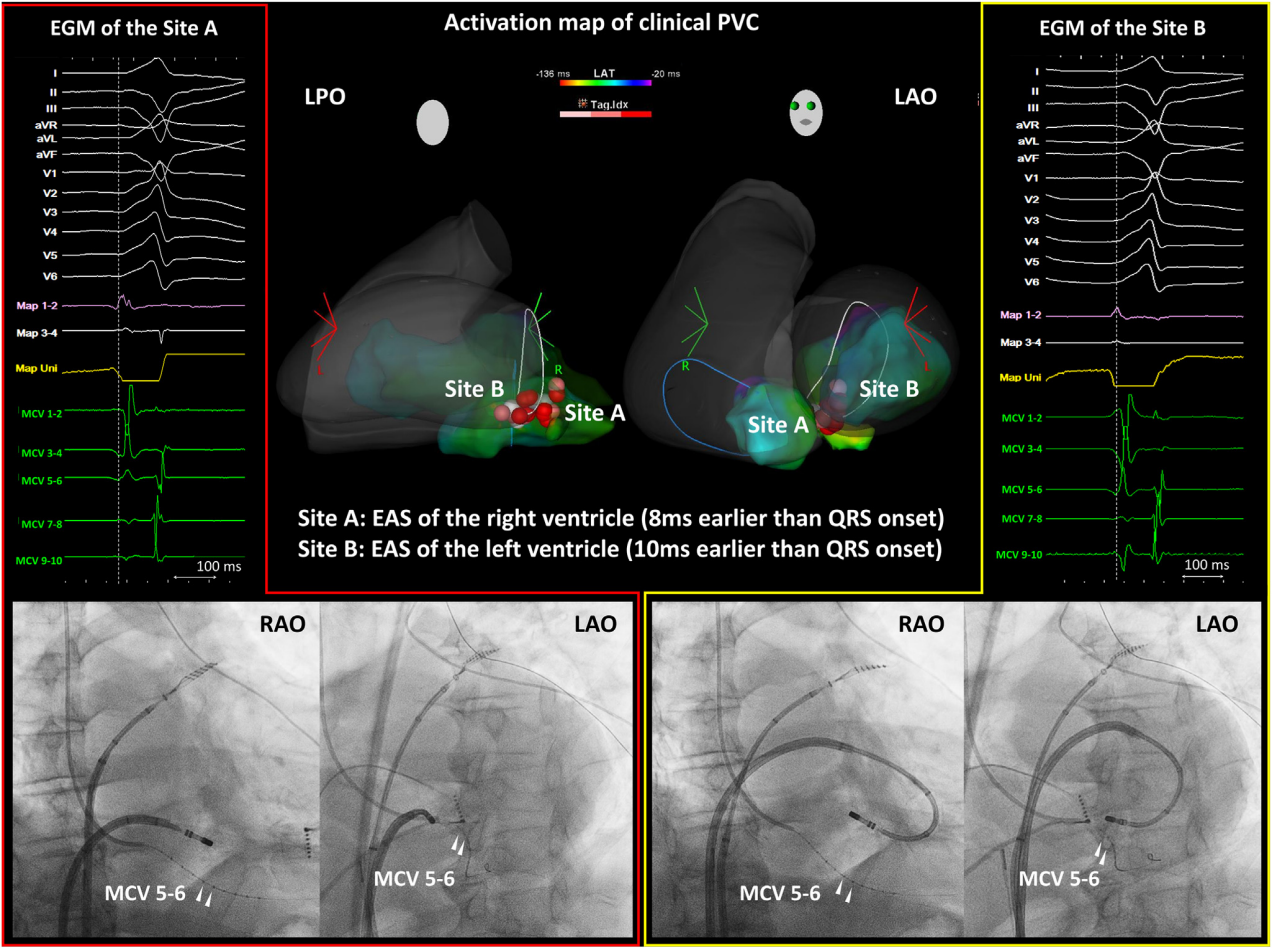
The electroanatomic mapping, intracardiac electrograms (EGMs), and fluoroscopic images of the radiofrequency (RF) energy application sites in case 2. The *red tag* indicates the RF energy application site. The local EGMs of the microelectrode catheter within the middle cardiac vein (MCV) did not precede the endocardial earliest activation site (EAS), and the timing of the right and left endocardial EASs was nearly identical, suggesting that the premature ventricular contraction (PVC) origin was located between the 2 EASs. RF energy was applied at both the right atrioventricular septum (*site A*) and left ventricular basal septum (*site B*). However, the occurrence of junctional rhythm and intermittent atrioventricular block during the procedure prevented the right-sided approach. Conversely, RF application from the left-sided EAS induced junctional rhythm, but did not cause an atrioventricular block. With careful monitoring, extended RF energy application from the left-sided EAS successfully eliminated the PVCs. Multiple tags were attached around the successful ablation site due to unstable breathing. LAO = left anterior oblique; LPO = left posterior oblique; RAO = right anterior oblique.

The electroanatomic mapping, intracardiac electrograms, and fluoroscopic images for this case are summarized in Figure 3. A total of 5 RF applications were delivered to the left-sided EAS, with a cumulative RF duration of approximately 360 seconds. Although PVCs recurred, their frequency decreased significantly, along with a reduction in the patient’s symptoms. A 24-hour Holter recording performed 3 months after the procedure revealed a substantial reduction in PVC burden (<1.0%). The patient reported significant symptom improvement and remained stable without requiring additional interventions.

## Discussion

We encountered 2 cases of PSP-LV PVCs in which the OTW microelectrode catheter, inserted into the MCV, was instrumental in identifying the targets for RF energy applications. In both cases, the electrograms recorded from the microelectrode catheter provided a valuable reference for examining the EAS.

The PSP-LV is located within a pyramidal space between the tricuspid and mitral annuli, serving as the confluence of all 4 cardiac chambers: the RA, left atrium, right ventricle, LV, and CS. Because the MCV lies centrally among these chambers, its electrograms can be a reliable reference for PSP-LV PVCs. However, placing conventional electrode catheters into the MCV can be challenging, especially when the MCV has a small diameter or steep curvature. The OTW microelectrode catheter, combined with the insertion technique described in Figure 1, significantly improved accessibility to the MCV. This technological advancement may enhance procedural success rates in the ablation of PSP-LV PVCs.

Given the anatomic complexity of the PSP-LV, accurate identification of the PVC origin is crucial. The OTW microelectrode catheter allows for precise identification of the EAS within the MCV by providing direct access to this challenging anatomic region. In addition, because the coronary venous system lies on the epicardial surface, electrograms recorded from the OTW microelectrode catheter in the MCV provide valuable epicardial insights. When integrated with the right and left endocardial electrograms, these epicardial recordings enhance our ability to estimate the PVC origin, particularly in cases with complex intramural or epicardial components. Although not necessary in all cases, the OTW microelectrode catheter may be beneficial when a PSP-LV PVC is suspected.

In case 1, initial RF energy applications suppressed the clinical PVC, but they recurred with a different morphology. The recurrent PVC exhibited a wider QRS complex and a larger MDI, suggesting an exit shift from the endocardium to the epicardium. The earliest electrogram of the recurrent PVC was recorded on the microelectrode catheter in the MCV, which preceded the QRS onset by 27 ms. This timing was earlier than any electrograms recorded on the right endocardium. Pacing at the epicardial EAS demonstrated excellent morphologic correlation with the recurrent PVC, suggesting that the PVC originated from this location. Conversely, in case 2, the earliest epicardial electrograms did not precede the endocardial electrograms, and the right and left endocardial electrograms exhibited comparably early activation. Pacing from the left endocardial EAS showed poor correlation with the clinical PVC, indicating an intramural origin of the PVC between the right and left endocardial EASs. These findings highlight the importance of integrating both epicardial and endocardial data to improve the estimation of the PVC origin.^4^

Despite its advantages, the OTW microelectrode catheter has certain limitations. First, catheter placement within the MCV may not always be feasible, particularly in cases with tortuous anatomy or a small MCV. Coronary angiography or venography can help delineate the MCV’s course and facilitate catheter placement. Second, although the OTW catheter can be integrated with impedance-based mapping systems, its lack of a magnetic sensor limits its compatibility with magnetic field–based electroanatomic mapping systems. Third, there is a potential risk of venous injury during guidewire advancement. However, using a floppy-tipped 0.014-inch guidewire minimizes this risk, and no complications were observed in our cases. Finally, beyond these catheter-specific limitations, RF energy application within the MCV presents additional challenges. Even after identifying a potential ablation target, delivering RF energy at this site requires careful consideration. Ensuring a low impedance and stable contact force is crucial, as these factors are critical for minimizing the risk of coronary artery injury during ablation within the coronary venous system.^6^ When RF ablation within the MCV is not feasible due to high impedance or unstable contact force, identifying the EAS—including those recorded from the MCV—can guide alternative strategies, such as an anatomic approach targeting the nearest cardiac chamber.

## Conclusion

We encountered 2 cases of PSP-LV PVCs in which RF energy applications were required from multiple chambers surrounding the crux of the heart. The 2.7F OTW microelectrode catheter inserted into the MCV proved to be an invaluable reference tool for estimating the origin of the PSP-LV PVCs, providing critical guidance for a safe and effective ablation strategy.

## Disclosures

The authors have no conflicts of interest to disclose.

## Appendix Supplementary Data

Supplementray Figure 1

Supplementray Figure 2
