# Supplementary material for: Over-the-wire microelectrode catheter in the middle cardiac vein for treating premature ventricular contractions from the posterior-superior process of the left ventricle
Source: HeartRhythm Case Rep. 2025 Apr 11;11(7):626–31. doi: 10.1016/j.hrcr.2025.04.004 (PMC12432841; doi:10.1016/j.hrcr.2025.04.004)
Supplement: Supplementray Figure 2 [file mmc2.docx]

**Supplementary Figure 2**


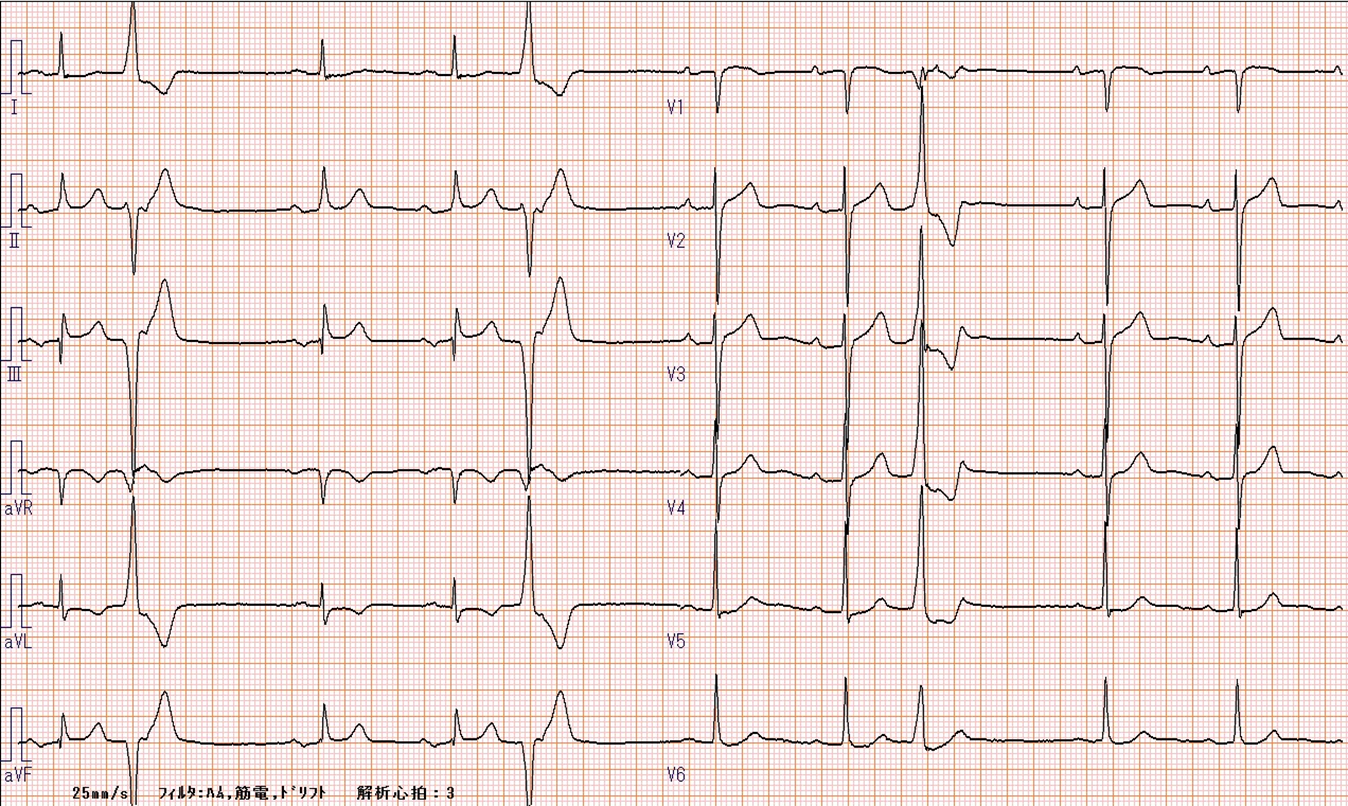


Preprocedural 12-lead electrocardiogram in Case 2 showing frequent premature ventricular contractions.
